# Genetic ancestry proportion influences risk of adverse events from tuberculosis treatment in Brazil

**DOI:** 10.1007/s00439-025-02809-y

**Published:** 2026-01-06

**Authors:** Jacqueline A. Piekos, Gustavo Amorim, Felipe Ridolfi, Marcelo Cordeiro-Santos, Afrânio L. Kritski, Marina C. Figueiredo, Bruno B. Andrade, Adalberto R. Santos, David W. Haas, Timothy R. Sterling, Valeria C. Rolla, Digna R. Velez Edwards

**Affiliations:** 1 Vanderbilt Genetics Institute, Vanderbilt University Medical Center, Nashville, TN USA; 2 Department of Biomedical Informatics, Vanderbilt University Medical Center, 2525 West End Ave, Suite 1150, Nashville, TN 37027 USA; 3 Division of Quantitative and Clinical Sciences, Department of Obstetrics and Gynecology, Vanderbilt University Medical Center, Nashville, TN USA; 4 Instituto Nacional de Infectologia Evandro Chagas, Fundação Oswaldo Cruz, Rio de Janeiro, Brazil; 5 Department of Biostatistics, Vanderbilt University Medical Center, Nashville, TN USA; 6 Division of Infectious Disease, Department of Medicine, Vanderbilt University Medical Center, Nashville, TN USA; 7 Fundação Medicina Tropical Dr. Heitor Vieira Dourado, Manaus, Brazil; 8 Universidade do Estado do Amazonas (UEA), Manaus, Brazil; 9 Faculdade de Medicina, Universidade Federal do Rio de Janeiro, Rio de Janeiro, Brazil; 10 Multinational Organization Network Sponsoring Translational and Epidemiological Research (MONSTER) Initiative, Bahia, Brazil; 11 Instituto Brasileiro Para Investigação da Tuberculose, Fundação Jose Silveira, Salvador, Bahia Brazil; 12 Instituto Goncalo Moniz, Fundação Oswaldo Cruz, Salvador, Bahia Brazil; 13 Escola Bahiana de Medicina e Saúde Publica (EBMSP), Salvador, Bahia Brazil; 14 Universidade Salvador (UNIFACS), Laureate Universities, Salvador, Bahia Brazil; 15 Faculdade de Tecnologia e Ciências (FTC), Salvador, Bahia Brazil; 16 Laboratory of Molecular Biology Applied to Mycobacteria, Instituto Oswaldo Cruz, Fundação Oswaldo Cruz, Rio de Janeiro, Brazil; 17 Department of Internal Medicine, Meharry Medical College, Nashville, TN USA

## Abstract

Tuberculosis (TB) treatment is highly effective, but response to therapy varies by geography and population subgroups. We assessed differences in TB treatment response in a representative and heterogeneous Brazilian population. We estimated genetic ancestry according to major genetic ancestry groups (African, European, and Amerindian) in the Regional Prospective Observational Research in Tuberculosis (RePORT)-Brazil cohort using ADMIXTURE software. RePORT-Brazil is an observational prospective cohort study of individuals with newly-diagnosed, culture-confirmed, pulmonary TB. Outcomes attributed to TB treatment included Grade 2 or higher adverse drug reaction (ADR), Grade 3 or higher ADR, hepatic ADR, and failure/recurrence. Genetic ancestry proportions were evaluated as predictors in univariate and multivariable logistic regression models for each outcome, and in stratified models for each genetic ancestry group. There were 930 pulmonary TB patients included in this study. In multivariable models we observed a decreased risk of Grade 2 + ADR when African ancestry proportion increased by 10% (Odds Ratio [OR] 0.41, 95% Confidence Interval [CI] 0.20–0.85) and an increased risk for Grade 2 + ADR with increasing European genetic ancestry (OR 2.33, 95% CI 1.14–4.76). In secondary analyses evaluating the interaction of HIV and genetic ancestry, we observed a statistically significant interaction between HIV and African genetic ancestry, but significantly decreased risk for Grade 2 + ADR with increasing African ancestry proportion. There were no associations with Amerindian genetic ancestry or for other treatment outcomes. In this Brazilian TB cohort, increased toxicity risk was associated with decreased African and increased European ancestry proportion.

**Supplementary Information:**

The online version contains supplementary material available at 10.1007/s00439-025-02809-y.

## Introduction

Tuberculosis (TB) is a global health problem that remains endemic in places throughout the world. According to the World Health Organization (WHO), Brazil is one of 30 high TB burden countries (World Health Organization 2024). In 2022, the incidence of new TB cases in Brazil was 36.3 cases per 100,000 population, while the mortality rate was 2.3 deaths per 100,000 population (Secretaria de Vigilancia em Saude e Ambiente 2023; Tavares et al. 2024). Over the last ten years, TB incidence has increased slightly in Brazil, while the mortality rate has remained steady at 2.2 deaths per 100,000 population (Silva Júnior et al. 2023).

In 2014, WHO presented its “End TB Strategy” with the goal to end the TB endemic worldwide by 2030. An important pillar of this strategy is to treat all TB patients with standard therapy: isoniazid, rifampin, pyrazinamide, and ethambutol for two months, followed by isoniazid and rifampin for four months (Nahid et al. 2016). In Brazil, about 70% of all TB patients are cured and recover from TB; the primary reason for lack of cure is nonadherence to TB treatment (Rabahi et al. 2017). The medications used to treat TB can often cause adverse drug reactions (ADR), such as metabolic and gastro-intestinal system disorders. One drug susceptible (DS)-TB cohort study in Brazil reported that almost 80% of their study participants reported at least one ADR during treatment, though 88% of the ADRs were in the mild/moderate (grade 1 or 2) category, and they could not all be attributed to TB treatment (Sant´ Anna et al. 2022). A previous study from RePORT-Brazil that recruited TB patients with newly diagnosed, culture confirmed, pulmonary TB found that 11% of study participants experienced an ADR that could be attributed to TB treatment (Ridolfi et al. 2024). TB-HIV coinfection greatly increases an individual’s risk for an ADR, due in large part to the combination of medications required to treat both TB and HIV.

One way in which ADRs can be reduced is through the use of pharmacogenomic precision medicine. Pharmacogenomics involves studying differences in genetic sequences that affect drug treatment in some way. The diversity of genetic variants within and between population groups can be informative regarding optimal treatment (Khoury et al. 2012). Genetic variation is not random, however; it is influenced by population structure. Population structure is the result of human migrations, isolations, and admixing over the course of history (Rosenberg et al. 2002; Ashraf and Galor 2013). An individual’s genetic ancestry reflects these movements, and influences the possible genetic variants that can be inherited. Genetic variation has been discovered within drug metabolizing enzymes, drug transporters, and drug receptors or targets that affect the pharmacokinetics and pharmacodynamics of a drug (Wilson et al. 2001; Wei et al. 2012; Yang et al. 2021). Understanding the relationship between genetic ancestry and pharmacodynamics can help advance precision medicine, and potentially decrease the risk of ADR, especially in highly-admixed populations (Micaglio et al. 2021; Pirmohamed 2023; Wei et al. 2012; Hernandez et al. 2020; Suarez-Kurtz 2005).

Precision medicine approaches for TB treatment have been previously undertaken and have mainly explored genetic variants within the *N-Acetyltransferase2* (*NAT2*) gene (Huang 2007; Sabbagh et al. 2008). Individuals with two slow acetylator alleles of the *NAT2* gene have been found to be at higher risk of ADR from anti-TB treatment (Zhang et al. 2018). We have also noted an association between *NAT2* genotype and TB treatment toxicity risk in RePORT-Brazil (Amorim et al. 2025). *NAT2* genotypes are estimated to account for 80% of the pharmacokinetic variability of isoniazid. (Sabbagh et al. 2008; Du et al. 2013; Kinzig-Schippers et al. 2005). In 2021, Verma et al. proposed a prototype assay for *NAT2* genotype determination with the goal of using it for dose-adjusted isoniazid treatment (2021). Other genetic factors that influence risk for ADR from anti-TB treatment are poorly understood. Research is ongoing to find biomarkers besides *NAT2* that affect anti-TB treatment outcomes. Using transcriptomics, researchers haves looked for biomarkers that effect TB treatment response (Thompson et al. 2017) and longevity of treatment (Heyckendorf et al. 2021). Exploring the relationships between genetic ancestry variation and pharmacogenomics has become an important area of research and has the potential to improve treatments for individuals (Yang et al. 2021; Lauschke and Ingelman-Sundberg 2020).

Our present study evaluated genetic ancestry differences in TB drug response—specifically toxicity and effectiveness outcomes—within a Brazilian TB cohort. The population of Brazil has high levels of genetic admixture primarily across European, African, and indigenous genetic ancestry groups, as well as high incidence and prevalence of TB (Saloum de Neves Manta et al. 2013; Galanter et al. 2012). In this study we characterized the genetic ancestry proportion of study participants and tested for associations between genetic ancestry and TB treatment outcomes.

## Methods

### Cohort description

The study population was from Regional Prospective Observational Research in Tuberculosis (RePORT)-Brazil, an observational prospective cohort study of individuals with newly-diagnosed, culture-confirmed, pulmonary tuberculosis and their close contacts (Arriaga et al. 2021). Participant enrollment occurred between June 2015 and June 2019 at five sites across three regions of Brazil. Three sites were in Rio de Janeiro (Instituto Nacional de Infectologia Evandro Chagas, Clínica de Saúde Rinaldo Delmare, Secretaria de Saúde de Duque de Caxias), one in Salvador (Instituto Brasileiro para Investigação da Tuberculose), and one in Manaus (Fundação Medicina Tropical Dr. Heitor Vieira Dourado). Characteristics of RePORT-Brazil have been described elsewhere; the study population is representative of all TB cases reported in Brazil (Arriaga et al. 2021). RePORT-Brazil participants were included in this study if they were ≥ 18 years old, provided samples for genetic testing and had culture-confirmed, drug-susceptible, pulmonary tuberculosis and received standard anti-TB therapy (isoniazid, rifampin, pyrazinamide, and ethambutol for two months, followed by isoniazid and rifampin for four months). Participants were followed for 24 months to assess TB treatment response and for TB recurrence. RePORT-Brazil excluded individuals who had previously received anti-TB therapy for ≥ 7 days, received > 7 days of fluoroquinolone therapy within 30 days prior to enrollment, were pregnant or breastfeeding, or planned to leave the region during follow-up.

All RePORT-Brazil study participants provided informed consent. The study was approved by the Institutional Review Boards at all study sites and at Vanderbilt University Medical Center.

### Data collection and variables

Clinical, demographic, and outcome (see below) data, as well as blood, urine, and plasma specimens, were collected longitudinally at four in-person study visits per RePORT-Brazil protocol. The following information was collected from participants at baseline: sex, age, body mass index (BMI), HbA1c, illicit drug use (never, previous, current), alcohol use (never, previous, current), and tobacco use (never, previous, current). All participants were tested for HIV at baseline unless already known to have HIV. Most participants were prescribed directly observed therapy (DOT) at treatment initiation.

Adverse drug reactions (ADR) were evaluated at all study visits and any other interaction with participants during TB treatment. The Division of AIDS (DAIDS) Table for Grading the Severity of Adult and Pediatric Adverse Events was used (2017); the Naranjo Algorithm was used to assess the probability of each ADR. Seven outcomes encompassing TB treatment toxicity, response and recurrence were evaluated in this study. For the outcomes grade 2 or higher ADR, grade 3 or higher ADR, and hepatic ADR, we tested all cases that had the outcome as well as a nested secondary set of cases that had the outcome attributed to TB treatment. When testing the outcomes attributed to TB treatment, participants who experienced the outcome but it was not attributed to TB treatment, were censored from analysis. Categories of physician-assigned attribution of causality were “possibly”, “likely”, or “definitely” related to TB treatment. The last outcome was treatment failure and/or recurrence. Treatment failure was defined as remaining sputum culture-positive or smear-positive at month 5 or later during treatment. Recurrence cases were defined as culture-confirmed tuberculosis or symptoms consistent with tuberculosis after the participant was considered cured or completed treatment. For all adverse outcomes, participants who experienced the outcome were compared to participants who did not.

### Genotyping

DNA was extracted from whole blood. For ancestry informative markers, genotyping was performed at Laboratório de Biologia Molecular Aplicada a Micobactérias in Fiocruz, using xMAP multiplexing technology (Luminex Corporation). There were 46 ancestry-informative markers (AIMs) selected for genotyping based on a previous analysis of a large and diverse Brazilian cohort (Saloum de Neves Manta et al. 2013). *NAT2* genotyping was performed by VANTAGE (Vanderbilt Technology for Advanced Genomics) using MassARRAY® iPLEX Gold (Agena Bioscience™, California, USA) and Taqman (ThermoFisher Scientific, Massachusetts, USA). Assay design is available upon request. We used *NAT2* (Ohno et al. 2000; Evans et al. 1960) gene variants (rs1801280, rs1799930, rs1799931, and rs1801279) to determine drug metabolism status, with intermediate (heterozygous at a single locus), slow (homozygous at any locus or heterozygous at 2 or more loci), and rapid (no variant allele) variants and used this variable as a covariate in analyses.

### Statistical analysis

Clinical and demographic characteristics were analyzed using a chi-square test for categorical and Wilcoxon ranksum test for continuous variables. ADMIXTURE (Alexander and Lange 2011) was used to determine genetic ancestry proportions of each participant based upon the selected AIMs (Manta et al. 2013). Using all references from the superpopulations of Europe, America, and Africa (n = 20) from the Human Genome Diversity Project (HGDP), (Bergström et al. 2020) we projected the 46 AIMs onto the reference populations and had unsupervised ADMIXTURE resolve the reference populations into K = 3 groups. K = 3 was determined to be the best number based on cross validation error analysis. ADMIXTURE calculated the allele frequencies of the AIMs for the reference populations and excluded reference populations that either could not be resolved with K = 3 means or were determined to be too heterogenous with genetic ancestry proportions below 60%. Fifteen of the reference populations were resolved into three superpopulations: European (EUR), African (AFR), and Amerindian (AME). Genetic ancestry proportions of the three superpopulations were then calculated for all samples.

Univariate and multivariable logistic regression models were used to evaluate the association between genetic ancestry proportion (African, European, Amerindian) and selected outcomes using the “rms” R package in R version 4.0.2 (Harrell 2023). We reported both adjusted and unadjusted models to assess the impact of covariates on effect size estimates. The association between each genetic ancestry proportion and outcomes was evaluated in separate models. We also performed secondary analyses assessing the interaction between HIV status and genetic ancestry proportion. Adjusted models included the following covariates: age, sex, BMI, study site, HIV status, baseline HbA1c, directly observed therapy (DOT), *NAT2* genotype, and usage of illicit drugs, alcohol, and tobacco. These a priori selected covariates were included based on their known association with TB treatment outcomes from previous epidemiological research (Barreto-Duarte et al. 2021). Covariate measurements were taken at baseline (enrollment). Outcomes were modeled using genetic ancestry and covariates, ancestry only, and covariates only. The reference group for region of enrollment was Rio de Janeiro and it included all three enrollment sites for that region. For *NAT2,* the rapid genotype was used as the reference for intermediate and slow metabolizers. Each ancestry proportion was evaluated as a continuous measure, with no transformations, and each was assessed in separate models. This approach has been used in prior published studies (Pérez-Jeldres et al. 2023; Parcha et al. 2022). Samples that did not pass genotyping quality control (QC) or had missing covariate or outcome information were excluded from the analysis.

## Results

### Population characteristics

Clinical and demographic characteristics, stratified by HIV status, of the RePORT cohort are given in Table 1A. The study population consisted of a total of 930 individuals; 19.4% were HIV positive. Amongst these individuals most were male (67%), never used tobacco (48%) or illicit drugs (66%), and had previously used alcohol (38%). Median and interquartile range [IQR] for age and BMI among HIV negative and HIV positive individuals was 36 years (IQR 25 to 50) and 35 years (IQR 28 to 42) for age and 20.2 (IQR 18.3 to 22.5) and 20.2 (1.84 to 22.5) for BMI, respectively. HIV status was found to significantly differ (p ≤ 0.05) between recruitment sites, sex, hemoglobin A1c, tobacco, alcohol, and illicit drug use. We also investigated the relationship between *NAT2* genotype and genetic ancestry and found them not to be correlated (range McFadden pseudo r^2^ 0.0007 to 0.003) (Supplemental Table 3).

**Table 1:** Population characteristics and adverse outcomes among participants in the RePORT Brazil cohort stratified by HIV status

A. Demographic characteristics and genetic ancestry estimates of the RePORT Brazil dataset, stratified by HIV status. Counts with percentages are given for categorical values and the median value with the inter-quartile range is given for continuous variables		
Variables	HIV Negative (N = 749)	HIV Positive (N = 181)
Categorical Variables		
Site, N (%)		
Rio de Janeiro (ref)	379 (51)	44 (24)
Salvador	229 (31)	2 (1)
Manaus	141 (19)	135 (75)
Sex, N (%)		
Males (ref)	480 (64)	139 (77)
Females	269 (36)	42 (23)
DOT, N (%)		
Not Prescribed (ref)	234 (31)	44 (24)
Prescribed	515 (69)	137 (76)
*NAT2* Genotype, N (%)		
Rapid (ref)	60 (8)	17 (9)
Intermediate	303 (40)	76 (42)
Slow	386 (52)	88 (49)
Tobacco Use, N (%)		
Never (ref)	373 (50)	71 (39)
Previous user	194 (26)	80 (44)
Current User	182 (24)	30 (17)
Alcohol Use, N (%)		
Never (ref)	136 (18.16)	12 (6.63)
Previous user	248 (33.11)	108 (59.67)
Current User	365 (48.73)	61 (33.70)
Illicit Drug Use, N (%)		
Never (ref)	530 (70.76)	84 (46.41)
Previous user	128 (17.09)	73 (40.33)
Current User	91 (12.15)	24 (13.26)
Continuous Variables		
BMI, Median kg/m^2^ (Q1, Q3)	20.2 (18.3, 22.5)	20.2 (18.4, 22.5)
Age, Median yrs (Q1, Q3)	36 (25, 50)	35 (28, 42)
HbA1c, Median (Q1, Q3)	5.8 (5.5, 6.4)	5.9 (5.4, 6.4)
Genetic Ancestry Proportion, Median (Q1, Q3)		
African	0.31 (0.17, 0.49)	0.19 (0.12, 0.29)
European	0.42 (0.28, 0.54)	0.41 (0.28, 0.53)
Amerindian	0.19 (0.13, 0.31)	0.33 (0.19, 0.49)

B. Counts for the number of individuals who experienced each adverse event investigated as an outcome and those who did not, stratified by HIV status				
	HIV Negative		HIV Positive	
Outcome	No Adverse Outcome	Adverse Outcome	No Adverse Outcome	Adverse Outcome
Grade 2 + ADR related to TB treatment	664	62	126	36
Grade 3 + ADR related to TB treatment	664	18	126	16
Hepatic ADR related to TB treatment	730	17	164	16
TB Treatment Failure/Recurrence	722	27	170	11

*HIV* human immunodeficiency virus, *ADR* adverse drug reaction, *TB* tuberculosis, *ref* reference, *DOT* directly observed therapy, *NAT2 N-acetyltransferase transferase 2*, *BMI* body mass index, *Q1* quartile 1, *Q3* quartile 3, *HbA1c* hemoglobin A1C

**Fig. 1:**
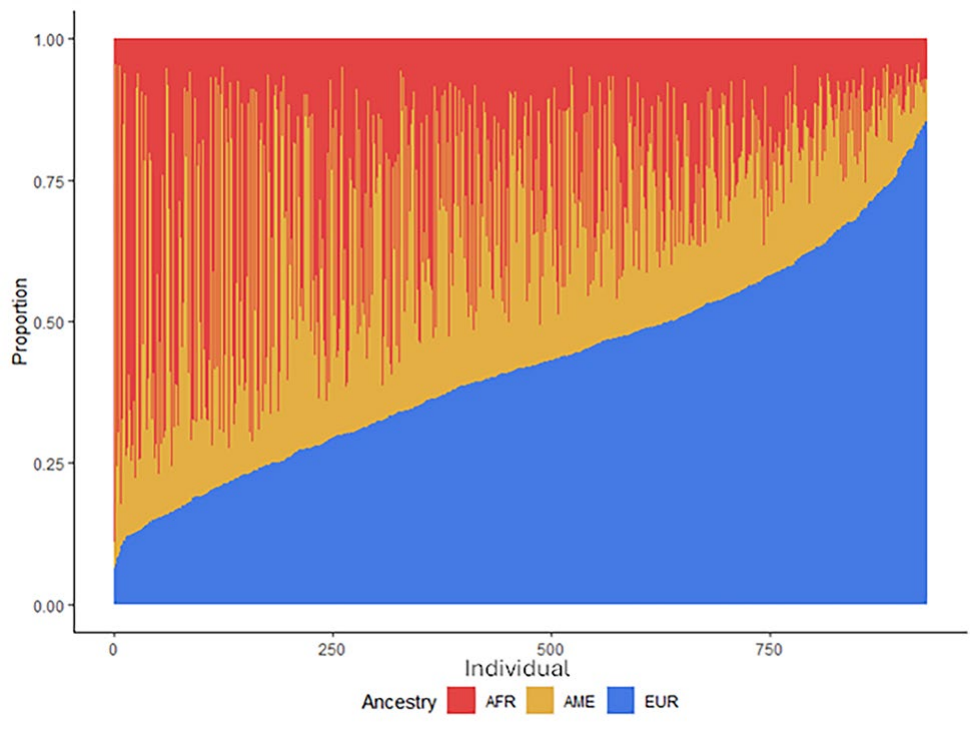
Ancestry proportions of the 930 genotyped study participants from the Brazil RePORT cohort. Subjects are represented on the x-axis (individual) and are ordered from lowest to highest proportion of European ancestry. Y-axis represents the proportion of genetic ancestry. AFR-African genetic ancestry; AME-Amerindian genetic ancestry; EUR-European genetic ancestry

The genetic ancestry proportions of all individuals in the cohort are summarized in Fig. 1. In this cohort, the median genetic ancestry proportions of an individual regardless of HIV status were 42% European, 32% African, and 26% Amerindian. When stratified on HIV status, the European ancestry proportion was similar between the groups, however HIV positive individuals had a higher average Amerindian genetic ancestry proportion and lower African genetic ancestry proportion. The trend was reversed for HIV negative individuals. (Table 1A).

Adverse outcomes related to TB treatment stratified by HIV status are given in Table 1B, with counts for all outcomes investigated provided in Supplemental Table 1. Participants who experienced the listed outcome were compared against participants who did not experience the outcome. Persons with grade 2 + ADR were censored from grade 3 + ADR analyses, and persons with ADRs that could not be directly attributed to TB medication were censored in the TB attributed outcomes. In the RePORT cohort, 15% of participants experienced at least one grade 2 + ADR event during the course of treatment, with 70% of those ADR events being attributed to TB medication. For the most general outcome, any grade 2 + ADR, demographic characteristics are given in Supplemental Table 2 stratified on ADR status. We found recruitment site (p = 2.82 × 10^–7^), DOT (p = 2.29 × 10^–11^), HIV status (p = 2.75 × 10^–10^), alcohol use (p = 5.00 × 10^–4^), illicit drug use (p = 0.03), hemoglobin A1c (p = 0.007), and African (p = 9.63X10^−5^)and European genetic (p = 0.05) ancestry proportions to be significantly associated with experiencing an ADR (Supplemental Table 2).

**Table 2:** Odds ratio of treatment outcomes for each 10% increase in ancestry proportion and significance level in unadjusted and adjusted logistic regression models

Outcome	Unadjusted				Adjusted			
Ancestry	OR (95% CI)	P-value	Pseudo-R^2^	AUC	OR (95% CI)	P-value	Pseudo-R^2^	AUC
Grade 2 + ADR related to TB Treatment								
African	0.34 (0.18–0.62)	5.0 × 10^–4^	0.03	0.62	0.41 (0.20–0.85)	0.02	0.23	0.80
European	2.84 (1.47–5.48)	0.002	0.02	0.59	2.33 (1.14–4.76)	0.02	0.23	0.80
Amerindian	1.36 (0.66–2.79)	0.40	0.002	0.52	0.95 (0.36–2.52)	0.92	0.22	0.79
Grade 3 + ADR related to TB Treatment								
African	0.40 (0.14–1.09)	0.07	0.02	0.59	1.05 (0.31–3.53)	0.94	0.24	0.86
European	1.00 (0.33–3.07)	1.00	0	0.49	0.78 (0.24–2.62)	0.69	0.24	0.86
Amerindian	3.02 (1.07–8.51)	0.04	0.02	0.58	1.32 (0.31–5.53)	0.71	0.24	0.86
Hepatic ADR related to TB Treatment								
African	1.12 (0.46–2.71)	0.80	0	0.50	1.63 (0.59–4.50)	0.35	0.18	0.80
European	1.03 (0.34–3.08)	0.96	0	0.51	0.73 (0.24–2.26)	0.59	0.18	0.81
Amerindian	0.80 (0.23–2.79)	0.73	0.001	0.52	0.68 (0.14–3.34)	0.63	0.18	0.80
Failure/Recurrence								
African	0.64 (0.27–1.53)	0.31	0.004	0.56	1.27 (0.42–3.86)	0.68	0.12	0.74
European	0.88 (0.31- 2.48)	0.81	0	0.51	1.36 (0.44–4.21)	0.60	0.12	0.75
Amerindian	2.07 (0.75–5.72)	0.16	0.007	0.57	0.50 (0.13–1.94)	0.32	0.12	0.75

*OR* odds ratio, *CI* confidence interval, *ADR* adverse drug reaction, *TB* Tuberculosis

Ancestry proportion was used as the main predictor in logistic regression modeling and adjusted models contained the covariates *NAT2* acetylator status, BMI, age, HbA1c, sex, DOT status, recruitment site, and smoking, alcohol, and drug usage. Odds ratios are given for 10% increase in ancestry proportion

### Genetic ancestry associations

**Fig. 2:**
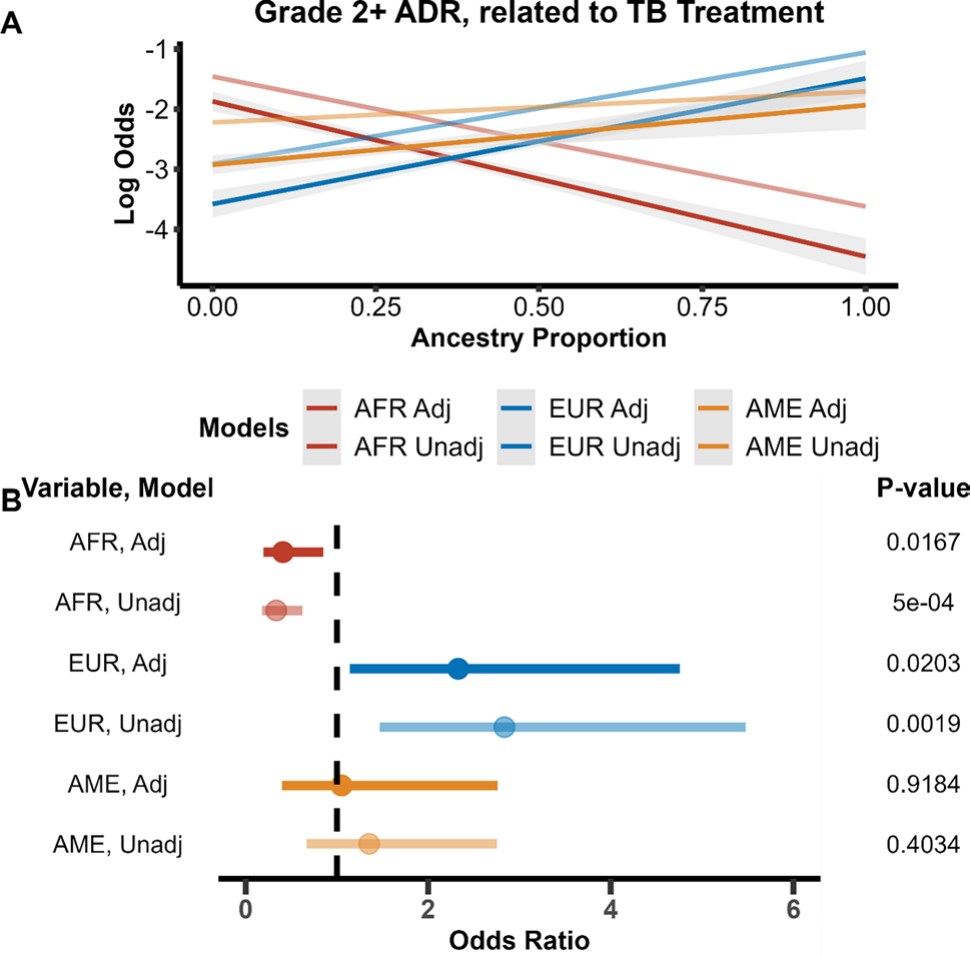
Logistic regression model results of outcome grade 2 + adverse drug reaction (ADR) related to tuberculosis (TB) using ancestry proportion as the main predictor. Unadjusted models are in darker colors, while the lighter colors are the models adjusted for BMI, age, HbA1c, sex, HIV status, DOT status, recruitment site, and smoking, alcohol, and drug usage. **A** Unadjusted and adjusted grade 2 + ADR related to TB modeled as log odds of having outcome as a function of ancestry proportion. African and European ancestry were found to be significant predictors for having a grade in the adjusted and unadjusted models and having a general adverse event in the unadjusted models. Increasing African ancestry genetic proportion decreases the risk of having an adverse drug event, related to TB or not, while increasing European ancestry genetic proportion increases the risk. **B** Odds ratios of ancestry variables and interaction terms of unadjusted and adjusted models plotted with their standard error. Significance level for variables is listed on the right. The dashed vertical line in Fig. 2B represents an odds ratio of 1

The outcome grade 2 + ADR attributed to TB treatment was significantly associated with African and European ancestry proportion, but not Amerindian ancestry proportion. (Fig. 2A) Increasing African genetic ancestry proportion by 10% was found to significantly decrease odds of the ADR outcome by 0.41 (95% confidence interval [CI]: 0.20–0.85, p = 0.02) in the adjusted model and 0.34 (95% CI: 0.18–0.62, p = 0.001) in unadjusted model. (Table 2) Increasing European ancestry by 10% was found to significantly increase odds of the ADR outcome by 2.33 (95% CI: 1.14–4.76, p = 0.02) in the adjusted model and 2.84 (95% CI: 1.47–5.48, p = 0.002) in the unadjusted model. (Table 2). For general grade 2 + ADR outcomes, all three ancestries were significantly associated with the outcome in the unadjusted models, but not in the adjusted models. African genetic ancestry was found to decrease odds for a grade 2 + ADR, while European and Amerindian genetic ancestry proportions increased the odds. Genetic ancestry being significantly associated with general grade 2 + ADR outcomes in unadjusted models but not in the adjusted model, suggests that the covariates included may have been responsible for the ADRs recorded. When grade 2 + ADR outcome was narrowed to specifically TB treatment causes, African and European genetic ancestry proportion was significant in adjusted and unadjusted models, suggesting that these two genetic ancestries played a role in ADR outcomes from TB medications.

Amerindian genetic ancestry was also significantly associated with grade 3 + ADR related to TB treatment in the unadjusted model only, (odds ratio [OR]: 3.02, 95% CI: 1.07–8.51, p = 0.04). (Table 2) The association did not hold in the adjusted model (p = 0.71). We did not detect any significant associations between African and European genetic ancestry proportions and experiencing a grade 3 + ADR, both general and attributed to TB medication. We did not detect any relationships between ancestry proportion and hepatic outcomes, both general and attributed to TB medication, and failure/recurrence. Supplemental Tables 4–10 provide the significance level of all variables for all seven investigated outcomes.

### Secondary interaction analyses of HIV status and genetic ancestry

**Fig. 3:**
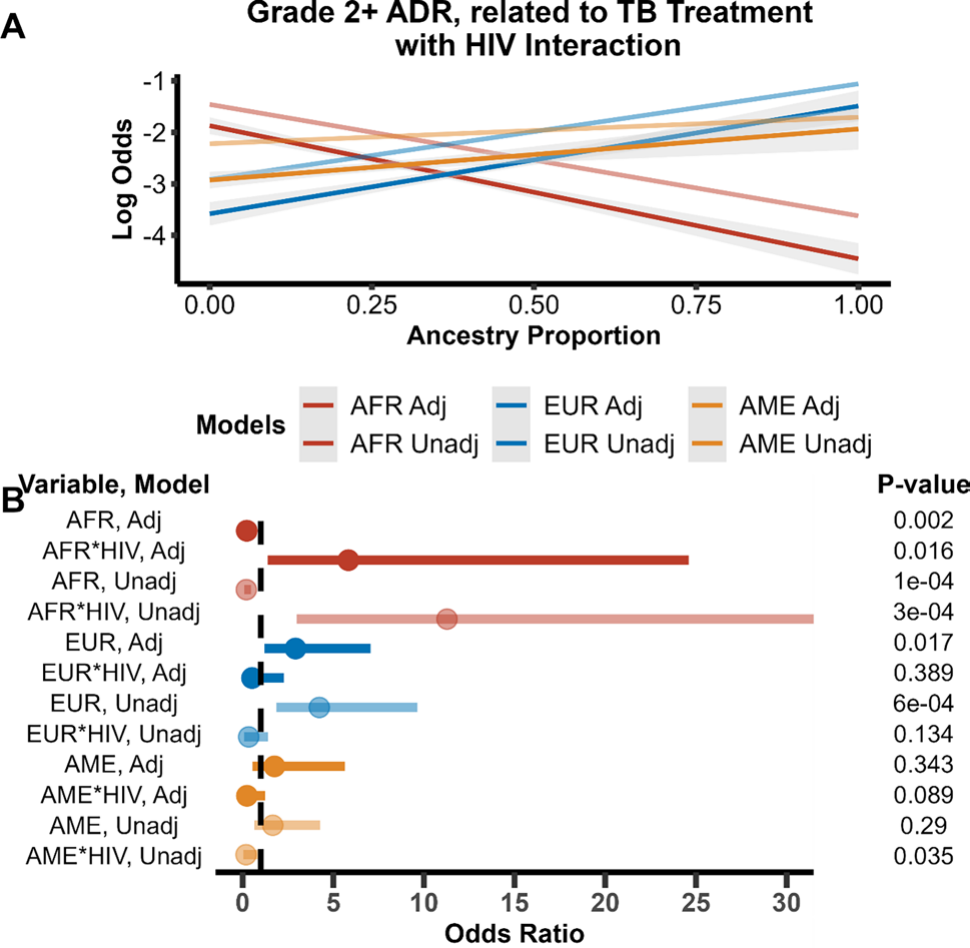
Logistic regression models for grade 2 + adverse drug reaction (ADR) related to TB stratified on HIV status. Adjusted models contain the covariates age, sex, BMI, treatment site, HgA1c, NAT2 genotype, directly observed therapy, and alcohol, smoking and illicit drug use status. **A** Unadjusted and adjusted logistic regression models of HIV negative individuals. Increasing African ancestry proportion lowers odds of a grade 2 + ADR related to TB while increasing European and Amerindian ancestry proportion increase odds. **B** Unadjusted and adjusted logistic regression models of HIV positive individuals. In this strata, increasing African and Amerindian ancestry proportion lowers odds of a grade 2 + ADR related to TB while increasing European ancestry proportion increase odds. The dashed vertical line in Fig. 2B represents an odds ratio of 1

We then tested if there was an interaction between HIV status and genetic ancestry for grade 2 + ADR related to TB treatment, as it is well-documented that treating HIV/TB co-infection is associated with a higher risk of ADR from treatment (Lönnroth et al. 2009; Barreto-Duarte et al. 2021; Pawlowski et al. 2012). HIV status and African genetic ancestry had a statistically significant interaction (p adjusted = 0.008) with, increasing African genetic ancestry proportion significantly associated with decreased grade 2 + ADR related to TB treatment. (Table 3, Fig. 3A). When we stratified analyses by HIV status, we observed an association with African ancestry and grade 2 + ADR amongst the HIV negative but not HIV positive individuals (Supplemental Table 11). The lack of association within HIV positive individuals may be due to small sample size in that strata.

**Table 3:** Grade 2 + adverse drug reaction related to tuberculosis treatment modeled using logistic regression with an interaction between ancestry proportions and HIV status

Variable	Unadjusted			Adjusted				
Interaction Term	OR (95% CI)	P-value	Pseudo-R^2^	OR (95% CI)	P-value		Pseudo-R^2^	
African	0.26 (0.13–0.52)	1 × 10^–4^	0.10	0.36 (0.17–0.76)		0.008		0.26
African*HIV	8.26 (2.64–25.79)	3 × 10^–4^		4.18 (1.22–14.37)		0.02		
European	3.14 (1.55–6.38)	0.002	0.08	2.17 (1.02–4.64)		0.05		0.26
European*HIV	0.26 (0.08–0.89)	0.03		0.41 (0.11–1.52)		0.18		
Amerindian	1.79 (0.80–4.02)	0.16	0.07	1.57 (0.57–4.28)		0.38		0.25
Amerindian*HIV	0.33 (0.10–1.14)	0.09		0.49 (0.13–1.87)		0.29		

*OR* odds ratio, *CI* confidence interval, *ADR* adverse drug reaction, *TB* Tuberculosis

Unadjusted models contain ancestry and interaction term. Ancestry proportion was used as the main predictor in logistic regression modeling and adjusted models contained the covariates *NAT2* acetylator status, BMI, age, HbA1c, sex, DOT status, recruitment site, and smoking, alcohol, and drug usage. Odds ratios are given for 10% increase in ancestry proportion

While there was evidence of interactions in univariate models there was no interaction between HIV status and European ancestry in multivariable analyses (Table 3, Fig. 3A). Amerindian genetic ancestry proportion and its interaction with HIV were not significant in unadjusted or adjusted models.

## Discussion

Here we observed how genetic ancestry influenced the outcomes of TB treatment in a cohort that is reflective of the TB population in Brazil. In this RePORT-Brazil study population, we found African genetic ancestry to be protective against grade 2 + ADR attributed to TB treatment while European genetic ancestry was associated with increased risk. This pattern was also seen with all grade 2 + ADR. Of note, *NAT2* genotype was not correlated with genetic ancestry in our analyses. A previous study of ADR in the TB registry in Brazil found self-determined populations descriptors Black and White to be protective against ADR when compared to Indigenous genetic ancestry (Barreto-Duarte et al. 2021). This study found African genetic ancestry to decrease risk for a grade 2 + ADR from TB treatments, exemplifying the differences between analyses of population descriptors and ancestry. The results of these two studies together suggest the health disparities observed in outcomes may be due to exogenous environmental variables that disproportionately affect self-reported Black individuals. We note that genetic ancestry differs from use of population descriptors and is a measure of inherited risk due to shared genetic factors (chromosomal segments) across individuals that have accumulated over time, often due to ancestors coming from the same geographic regions. We cannot conclude what effect genetic ancestry has biologically on TB drug toxicity; however, the associations we see with genetic ancestry would suggest that a portion of TB drug toxicity is heritable and may be similar among individuals with ancestry from the same geographic region.

The average genetic ancestry observed in the cohort was split almost evenly amongst European, African, and Amerindian genetic ancestry. We observed that individuals from the Manaus site had a slightly higher proportion of Amerindian ancestry on average than individuals from Rio de Janeiro and Salvador. This aligns with previous studies in other South American populations that have found individuals in urban areas to have higher proportions of European and African genetic ancestry, and individuals living more inland have a higher proportion of Amerindian genetic ancestry (Ruiz-Linares et al. 2014; Gontijo et al. 2014; Homburger et al. 2015). The Manaus site is further inland in Brazil, where there has been less admixture between the indigenous populations who originally inhabited the area, and incoming settlers.

TB is known to be a common comorbidity among individuals living with HIV. We found that an increasing proportion of African genetic ancestry was associated with a decreasing risk of grade 2 + ADR due to TB treatment. There was an interaction between HIV and African ancestry, but the relationship still held after accounting for HIV (Table 3). However, in an analysis stratified by HIV status, African ancestry proportion was not significantly associated with the risk of grade 2 + ADR related to TB treatment in persons with HIV (Supplemental Table 11). This lack of association may be due at least in part to the relatively small sample size among persons with HIV and thus should be further evaluated in future studies. Also, it is possible that the interaction could go in both directions: ancestry could modify the effect of HIV on outcome, and HIV could modify the effect of ancestry on outcome. However, this is not well-understood.

There were several limitations of our study.First, there were relatively few grade 3 or higher ADR, which precluded an assessment for a possible association between ancestry and grade 3 or higher toxicity. Second, the sample size of persons with HIV was also relatively small, which contributed to decreased statistical power to detect associations in this population, as well as possible interactions between HIV and ancestry on toxicity risk. Third, the definitions of the genetic ancestry groups were averages, and represented a combination of genetic groupings. Use of continental ancestry groups meant that information on intercontinental variation was not included. Intercontinental variation is important, particularly for African populations (Sabbagh et al. 2011). We also note that at the time of this study we only had AIMs available to assess ancestry proportions and no GWAS data from which to estimate global ancestry. While the lack of global ancestry estimates may limit our ability to capture higher orders of variances due to other smaller represented ancestry groups, with the use of validated AIMs we were able to capture the three major ancestry groups represented in our data.

To our knowledge this is the first study to evaluate the relationship between genetic ancestry and TB treatment outcomes. A previous study found that self-determined population descriptors (Black or Brown compared to White), was associated with an increased risk of adverse events from TB treatment (Viana et al. 2016). In this study, by assessing genetic ancestry, we were able to assess contributions of genetic ancestry rather than population descriptors regarding the outcomes studied. We found that increased African genetic ancestry proportion was protective against adverse TB treatment toxicity outcomes, and increased European ancestry proportion was associated with increased toxicity risk. These associations held after accounting for interactions with HIV. These data suggest that differences in TB treatment outcomes may be due in part to differences in genetic ancestry.

## Supplementary Information

Below is the link to the electronic supplementary material.

Supplementary Material 1

## Data Availability

Data is provided within the manuscript or supplementary and figure files.
