# Supplementary material for: Genetic ancestry proportion influences risk of adverse events from tuberculosis treatment in Brazil
Source: Hum Genet. 2026 Jan 6;145(1):10. doi: 10.1007/s00439-025-02809-y (PMC12774987; doi:10.1007/s00439-025-02809-y)
Supplement: Supplementary file 1 — Supplementary Material 1 [file 439_2025_2809_MOESM1_ESM.docx]

**Genetic ancestry proportion influences risk of adverse events from tuberculosis treatment in Brazil**

**Human Genetics**

Jacqueline A. Piekos,^1-3^ Gustavo Amorim,^5^ Felipe Ridolfi,^4,6^ Marcelo Cordeiro-Santos,^7,8^ Afrânio L. Kritski,^9^ Marina C. Figueiredo,^6^ Bruno B. Andrade,^10-15^ Adalberto R. Santos,^16^ David W. Haas,^6,17^ Timothy R. Sterling,^6†^ Valeria C. Rolla,^4†^ Digna R. Velez Edwards,^2,3†*^ and on behalf of the Regional Prospective Observational Research in Tuberculosis (RePORT)-Brazil consortium

^1^Vanderbilt Genetics Institute, Vanderbilt University Medical Center, Nashville, TN, USA

^2^Department of Biomedical Informatics, Vanderbilt University Medical Center, Nashville, TN, USA

^3^Division of Quantitative and Clinical Sciences, Department of Obstetrics and Gynecology, Vanderbilt University Medical Center, Nashville, TN, USA

^4^Instituto Nacional de Infectologia Evandro Chagas, Fundação Oswaldo Cruz, Rio de Janeiro, Brazil

^5^Department of Biostatistics, Vanderbilt University Medical Center, Nashville, TN, USA

^6^Division of Infectious Disease, Department of Medicine, Vanderbilt University Medical Center, Nashville, TN, USA

^7^Fundação Medicina Tropical Dr. Heitor Vieira Dourado, Manaus, Brazil

^8^Universidade do Estado do Amazonas (UEA), Manaus, Brazil

^9^Faculdade de Medicina, Universidade Federal do Rio de Janeiro, Rio de Janeiro, Brazil

^10^Multinational Organization Network Sponsoring Translational and Epidemiological Research (MONSTER) Initiative, Bahia, Brazil

^11^Instituto Brasileiro para Investigação da Tuberculose, Fundação Jose Silveira, Salvador, Bahia, Brazil

^12^Instituto Goncalo Moniz, Fundação Oswaldo Cruz, Salvador, Bahia, Brazil

^13^Escola Bahiana de Medicina e Saúde Publica (EBMSP), Salvador, Bahia, Brazil

^14^Universidade Salvador (UNIFACS), Laureate Universities, Salvador, Bahia, Brazil

^15^Faculdade de Tecnologia e Ciências (FTC) Salvador, Bahia, Brazil

^16^Laboratory of Molecular Biology Applied to Mycobacteria, Instituto Oswaldo Cruz, Fundação Oswaldo Cruz, Rio de Janeiro, Brazil

^17^ Department of Internal Medicine, Meharry Medical College, Nashville, TN, USA

#RePORT-Brazil Consortium members include:

- Aline Benjamin and Flavia M. Sant’Anna
  - Instituto Nacional de Infectologia Evandro Chagas, Fiocruz, Rio de Janeiro, Brazil
- Solange Cavalcante, Betina Durovni, Jamile Garcia de Oliveira and João Marine
  - Clínica de Saúde Rinaldo Delmare, Rio de Janeiro, Brazil
- Adriana Rezende and Anna Cristina Carvalho
  - Secretaria de Saúde de Duque de Caxias, Rio de Janeiro, Brazil
- Michael Rocha and Betânia Nogueira
  - Instituto Brasileiro para Investigação da Tuberculose, Fundação José Silveira, Salvador, Brazil
- Alexandra Brito and Renata Spener
  - Fundação Medicina Tropical Dr. Heitor Vieira Dourado, Manaus, Brazil
- Megan Turner, James Jaworski
  - Vanderbilt University Medical Center, Nashville, USA

*Corresponding Author:

Digna R Velez Edwards, PhD, MS

Email: [digna.r.velez.edwards@vumc.org](mailto:digna.r.velez.edwards@vumc.org)

# **Supplemental Tables**

**Supplemental Table 1.**

| Outcome | Total N | Did not have the Event | Had the Event |
| --- | --- | --- | --- |
| All ADR Grade 2 and higher | 930 | 790 | 140 |
| ADR Grade 2 and higher related to TB treatment | 888 | 790 | 98 |
| All ADR Grade 3 and higher | 848 | 790 | 58 |
| ADR Grade 3 and higher related to TB treatment | 824 | 790 | 34 |
| All Hepatic ADR | 930 | 894 | 36 |
| Hepatic ADR related to TB treatment | 927 | 894 | 33 |
| TB treatment Failure or Recurrence | 930 | 892 | 38 |

ADR: adverse drug reaction

**Supplemental Table 2:** Clinical and demographic characteristics of the study population according to those who developed Grade 2+ adverse drug reactions and those who did not. Categorical data are presented as number (percentage); continuous variables are presented as median and interquartile range. P-values are from the chi-square test for categorical variables and the Wilcoxon Rank Sum test for continuous variables.

| *Categorical Variable* | Grade 2+ ADR  N = 140 | % | No Grade 2+ ADR  N = 790 | % | P |
| --- | --- | --- | --- | --- | --- |
| Site |  |  |  |  |  |
| Rio | 77 | 0.55 | 346 | 0.44 | 2.82E-07 |
| Salvador | 9 | 0.06 | 222 | 0.28 |  |
| Manaus | 54 | 0.39 | 222 | 0.28 |  |
| Sex |  |  |  |  |  |
| Male | 95 | 0.68 | 524 | 0.66 | 0.80 |
| Female | 45 | 0.32 | 266 | 0.34 |  |
| DOT |  |  |  |  |  |
| No | 8 | 0.06 | 270 | 0.34 | 2.39E-11 |
| Yes | 132 | 0.94 | 520 | 0.66 |  |
| HIV |  |  |  |  |  |
| Negative | 85 | 0.61 | 664 | 0.84 | 2.75E-10 |
| Positive | 55 | 0.39 | 126 | 0.16 |  |
| *NAT2* |  |  |  |  |  |
| Rapid | 6 | 0.04 | 71 | 0.09 | 0.06 |
| Intermediate | 52 | 0.37 | 327 | 0.41 |  |
| Slow | 82 | 0.56 | 392 | 0.50 |  |
| Smoking |  |  |  |  |  |
| Never | 58 | 0.41 | 386 | 0.49 | 0.09 |
| Prior | 52 | 0.37 | 222 | 0.28 |  |
| Current | 30 | 0.21 | 182 | 0.23 |  |
| Alcohol |  |  |  |  |  |
| Never | 24 | 0.17 | 124 | 0.16 | 0.0005 |
| Prior | 72 | 0.51 | 284 | 0.36 |  |
| Current | 44 | 0.31 | 382 | 0.48 |  |
| Drug use |  |  |  |  |  |
| Never | 93 | 0.66 | 521 | 0.66 | 0.03 |
| Prior | 38 | 0.27 | 163 | 0.21 |  |
| Current | 9 | 0.06 | 106 | 0.13 |  |
| *Continuous Variables* |  |  |  |  |  |
| BMI | 20.4 | (18.6, 22.4) | 20.1 | (18.3, 22.5) | 0.46 |
| Age | 38 | (26, 49) | 35 | (25, 49) | 0.14 |
| HbA1c | 5.9 | (5.5, 6.4) | 5.8 | (5.4, 6.4) | 0.007 |
| Ancestry |  |  |  |  |  |
| AFR | 0.20 | (0.12, 0.37) | 0.29 | (0.16, 0.47) | 9.63E-05 |
| EUR | 0.44 | (0.3, 0.59) | 0.41 | (0.28, 0.54) | 0.05 |
| AME | 0.23 | (0.14, 0.38) | 0.21 | (0.13, 0.34) | 0.06 |

ADR: adverse drug reaction

DOT: directly-observed therapy

NAT2: N-acetyl transferase 2

BMI: body mass index

HbA1c: hemoglobin A1c

AFR: African ancestry

EUR: European ancestry

AME: Amerindian ancestry

**Supplemental Table 3:**

**A)** Correlation matrix of the three ancestry group proportions and *NAT2* genotype level. *NAT2* was modeled according to metabolizer status.

|  | AFR | EUR | AME | *NAT2* |
| --- | --- | --- | --- | --- |
| AFR | 1 | -0.53 | -0.54 | 0.07 |
| EUR | -0.53 | 1 | -0.25 | 0.02 |
| AME | -0.54 | -0.25 | 1 | -0.05 |
| *NAT2* | 0.07 | 0.02 | -0.05 | 1 |

**B)** Significance levels of the correlation measures between the three ancestry groups and *NAT2* genotype level. *NAT2* was modeled according to metabolizer status.

|  | AFR | EUR | AME | *NAT2* |
| --- | --- | --- | --- | --- |
| AFR |  | 0 | 0 | 0.05 |
| EUR | 0 |  | 0 | 0.53 |
| AME | 0 | 0 |  | 0.1 |
| *NAT2* | 0.05 | 0.53 | 0.1 |  |

**C)**

|  | *NAT2* (1=slow) | |  |
| --- | --- | --- | --- |
| Ancestry | **0** | **1** | **McFadden pseudo-r2** |
| AFR | 0.31 | 0.32 | 0.0007 |
| EUR | 0.41 | 0.42 | 0.0005 |
| AME | 0.28 | 0.26 | 0.003 |

NAT2: N-acetyl transferase 2

AFR: African ancestry

EUR: European ancestry

AME: Amerindian ancestry

**Supplemental Table 4:** Covariate results of logistic regression modeling of any grade 2 or higher adverse drug reaction (ADR) using ancestry proportion as the main predictor.

| Ancestry | Variable | Odds Ratio | Lower CI | Upper CI | P-value |
| --- | --- | --- | --- | --- | --- |
| African | Salvador | 0.30 | 0.14 | 0.64 | 0.002 |
| African | Manaus | 0.66 | 0.38 | 1.14 | 0.14 |
| African | Sex, female | 0.96 | 0.61 | 1.51 | 0.85 |
| African | HIV+ | 3.01 | 1.82 | 4.98 | <0.0001 |
| African | BMI | 1.04 | 0.98 | 1.10 | 0.18 |
| African | Age | 1.01 | 0.99 | 1.03 | 0.06 |
| African | Smoke, used to | 1.15 | 0.67 | 1.97 | 0.61 |
| African | Smoke, current | 1.56 | 0.84 | 2.87 | 0.16 |
| African | Alcohol, used to | 1.21 | 0.65 | 2.28 | 0.55 |
| African | Alcohol, current | 0.70 | 0.36 | 1.36 | 0.29 |
| African | HbA1c | 0.85 | 0.75 | 0.97 | 0.02 |
| African | DOT, yes | 8.76 | 4.13 | 18.61 | <0.0001 |
| African | Illicit drug use, prior | 0.84 | 0.48 | 1.47 | 0.54 |
| African | Illicit drug use, current | 0.41 | 0.18 | 0.97 | 0.04 |
| African | NAT2, intermediate | 1.97 | 0.77 | 5.01 | 0.16 |
| African | NAT2, slow | 2.89 | 1.15 | 7.25 | 0.02 |
| European | Salvador | 0.28 | 0.13 | 0.60 | 0.001 |
| European | Manaus | 0.84 | 0.50 | 1.40 | 0.50 |
| European | Sex, female | 0.96 | 0.61 | 1.50 | 0.85 |
| European | HIV+ | 2.92 | 1.79 | 4.87 | <0.0001 |
| European | BMI | 1.04 | 0.99 | 1.11 | 0.15 |
| European | Age | 1.01 | 1.00 | 1.03 | 0.07 |
| European | Smoke, used to | 1.12 | 0.70 | 1.91 | 0.68 |
| European | Smoke, current | 1.53 | 0.83 | 2.80 | 0.17 |
| European | Alcohol, prior | 1.22 | 0.65 | 2.30 | 0.53 |
| European | Alcohol, current | 0.70 | 0.36 | 1.36 | 0.30 |
| European | HbA1c | 0.85 | 0.75 | 0.97 | 0.02 |
| European | DOT, yes | 8.84 | 4.16 | 18.78 | <0.0001 |
| European | Illicit drug use, prior | 0.83 | 0.48 | 1.45 | 0.52 |
| European | Illicit drug use, current | 0.41 | 0.17 | 0.9 | 0.04 |
| European | NAT2, intermediate | 1.98 | 0.78 | 5.05 | 0.15 |
| European | NAT2, slow | 2.88 | 1.15 | 7.22 | 0.02 |
| Amerindian | Salvador | 0.27 | 0.13 | 0.57 | 0.0006 |
| Amerindian | Manaus | 0.76 | 0.42 | 1.39 | 0.38 |
| Amerindian | Sex, female | 0.98 | 0.62 | 1.53 | 0.91 |
| Amerindian | HIV+ | 2.94 | 1.7 | 4.85 | <0.0001 |
| Amerindian | BMI | 1.05 | 0.99 | 1.11 | 0.14 |
| Amerindian | Age | 1.02 | 1.00 | 1.0 | 0.05 |
| Amerindian | Smoke, prior | 1.3 | 0.6 | 1.9 | 0.66 |
| Amerindian | Smoke, current | 1.52 | 0.83 | 2.8 | 0.17 |
| Amerindian | Alcohol, prior | 1.19 | 0.64 | 2.23 | 0.59 |
| Amerindian | Alcohol, current | 0.67 | 0.35 | 1.30 | 0.24 |
| Amerindian | HbA1c | 0.85 | 0.76 | 0.97 | 0.01 |
| Amerindian | DOT, yes | 8.65 | 4.08 | 18.36 | <0.0001 |
| Amerindian | Illicit drug use, prior | 0.83 | 0.48 | 1.45 | 0.51 |
| Amerindian | Illicit drug use, current | 0.41 | 0.18 | 0.9 | 0.04 |
| Amerindian | NAT2, intermediate | 1.9 | 0.77 | 4.99 | 0.16 |
| Amerindian | NAT2, slow | 2.87 | 1.15 | 7.19 | 0.02 |

**Supplemental Table 5:** Covariate results of logistic regression modeling of grade 2 or higher adverse drug reactions related to TB treatment using ancestry proportion as the main predictor.

| Ancestry | Variable | Odds Ratio | Lower CI | Upper CI | P-value |
| --- | --- | --- | --- | --- | --- |
| African | Salvador | 0.40 | 0.18 | 0.88 | 0.02 |
| African | Manaus | 0.47 | 0.24 | 0.90 | 0.02 |
| African | Sex, female | 0.97 | 0.57 | 1.62 | 0.90 |
| African | HIV+ | 2.80 | 1.56 | 5.00 | 0.0005 |
| African | BMI | 1.07 | 1.00 | 1.14 | 0.04 |
| African | Age | 1.02 | 1.00 | 1.03 | 0.07 |
| African | Smoke, prior | 0.85 | 0.46 | 1.57 | 0.60 |
| African | Smoke, current | 1.00 | 0.50 | 2.04 | 1.00 |
| African | Alcohol, prior | 1.79 | 0.86 | 3.71 | 0.12 |
| African | Alcohol, current | 0.88 | 0.39 | 1.89 | 0.70 |
| African | HbA1c | 0.77 | 0.65 | 0.92 | 0.004 |
| African | DOT, yes | 6.15 | 2.86 | 13.23 | <0.0001 |
| African | Illicit drug use, prior | 0.95 | 0.50 | 1.81 | 0.88 |
| African | Illicit drug use, current | 0.53 | 0.20 | 1.44 | 0.21 |
| African | NAT2, intermediate | 1.84 | 0.60 | 5.61 | 0.28 |
| African | NAT2, slow | 3.17 | 1.06 | 9.48 | 0.04 |
| European | Salvador | 0.38 | 0.17 | 0.82 | 0.01 |
| European | Manaus | 0.68 | 0.37 | 1.24 | 0.21 |
| European | Sex, female | 0.96 | 0.57 | 1.61 | 0.88 |
| European | HIV+ | 2.73 | 1.53 | 4.87 | 0.0006 |
| European | BMI | 1.08 | 1.01 | 1.15 | 0.03 |
| European | Age | 1.01 | 1.00 | 1.03 | 0.11 |
| European | Smoke, prior | 0.81 | 0.4 | 1.50 | 0.50 |
| European | Smoke, current | 0.98 | 0.48 | 1.99 | 0.96 |
| European | Alcohol, prior | 1.82 | 0.88 | 3.77 | 0.11 |
| European | Alcohol, current | 0.85 | 0.39 | 1.86 | 0.69 |
| European | HbA1c | 0.77 | 0.65 | 0.92 | 0.004 |
| European | DOT, yes | 6.28 | 2.91 | 13.54 | <0.0001 |
| European | Illicit drug use, prior | 0.94 | 0.50 | 1.78 | 0.85 |
| European | Illicit drug use, current | 0.51 | 0.19 | 1.39 | 0.19 |
| European | NAT2, intermediate | 1.88 | 0.62 | 5.71 | 0.27 |
| European | NAT2, slow | 3.14 | 1.05 | 9.37 | 0.04 |
| Amerindian | Salvador | 0.34 | 0.16 | 0.73 | 0.01 |
| Amerindian | Manaus | 0.65 | 0.33 | 1.31 | 0.23 |
| Amerindian | Sex, female | 1.00 | 0.60 | 1.66 | 0.99 |
| Amerindian | HIV+ | 2.67 | 1.51 | 4.75 | 0.0008 |
| Amerindian | BMI | 1.08 | 1.01 | 1.15 | 0.02 |
| Amerindian | Age | 1.02 | 1.00 | 1.03 | 0.06 |
| Amerindian | Smoke, prior | 0.82 | 0.45 | 1.51 | 0.53 |
| Amerindian | Smoke, current | 0.99 | 0.49 | 2.00 | 0.97 |
| Amerindian | Alcohol, prior | 1.72 | 0.83 | 3.54 | 0.14 |
| Amerindian | Alcohol, current | 0.80 | 0.37 | 1.73 | 0.56 |
| Amerindian | HbA1c | 0.76 | 0.64 | 0.91 | 0.003 |
| Amerindian | DOT, yes | 6.13 | 2.85 | 13.20 | <0.0001 |
| Amerindian | Illicit drug use, prior | 0.92 | 0.49 | 1.74 | 0.81 |
| Amerindian | Illicit drug use, current | 0.51 | 0.19 | 1.38 | 0.18 |
| Amerindian | NAT2, intermediate | 1.84 | 0.61 | 5.58 | 0.28 |
| Amerindian | NAT2, slow | 3.12 | 1.05 | 9.28 | 0.04 |

**Supplemental Table 6:** Covariate results of logistic regression modeling of any grade 3 or higher ADR outcome using ancestry proportion as the main predictor.

| Ancestry | Variable | Odds Ratio | Lower CI | Upper CI | P-value |
| --- | --- | --- | --- | --- | --- |
| African | Salvador | 0.14 | 0.03 | 0.62 | 0.01 |
| African | Manaus | 0.42 | 0.19 | 0.95 | 0.04 |
| African | Sex, female | 1.03 | 0.54 | 1.98 | 0.93 |
| African | HIV+ | 5.48 | 2.64 | 11.40 | <0.0001 |
| African | BMI | 1.01 | 0.93 | 1.10 | 0.75 |
| African | Age | 1.03 | 1.01 | 1.06 | 0.003 |
| African | Smoke, prior | 1.40 | 0.64 | 3.05 | 0.40 |
| African | Smoke, current | 1.04 | 0.41 | 2.62 | 0.94 |
| African | Alcohol, prior | 0.49 | 0.20 | 1.20 | 0.12 |
| African | Alcohol, current | 0.58 | 0.23 | 1.44 | 0.24 |
| African | HbA1c | 0.95 | 0.80 | 1.12 | 0.53 |
| African | DOT, yes | 13.67 | 3.23 | 57.95 | 0.0004 |
| African | Illicit drug use, prior | 0.93 | 0.41 | 2.12 | 0.86 |
| African | Illicit drug use, current | 0.75 | 0.24 | 2.40 | 0.63 |
| African | NAT2, intermediate | 5.08 | 0.66 | 39.42 | 0.12 |
| African | NAT2, slow | 5.12 | 0.67 | 39.35 | 0.12 |
| European | Salvador | 0.14 | 0.03 | 0.61 | 0.01 |
| European | Manaus | 0.46 | 0.22 | 0.99 | 0.05 |
| European | Sex, female | 1.03 | 0.54 | 1.98 | 0.93 |
| European | HIV+ | 5.44 | 2.62 | 11.29 | <0.0001 |
| European | BMI | 1.02 | 0.93 | 1.10 | 0.73 |
| European | Age | 1.03 | 1.01 | 1.06 | 0.003 |
| European | Smoke, prior | 1.39 | 0.64 | 3.03 | 0.40 |
| European | Smoke, current | 1.04 | 0.41 | 2.62 | 0.94 |
| European | Alcohol, prior | 0.49 | 0.20 | 1.20 | 0.12 |
| European | Alcohol, current | 0.57 | 0.23 | 1.43 | 0.23 |
| European | HbA1c | 0.95 | 0.80 | 1.12 | 0.53 |
| European | DOT, yes | 13.78 | 3.25 | 58.46 | 0.0004 |
| European | Illicit drug use, prior | 0.93 | 0.41 | 2.12 | 0.86 |
| European | Illicit drug use, current | 0.75 | 0.23 | 2.37 | 0.62 |
| European | NAT2, intermediate | 5.11 | 0.66 | 39.62 | 0.12 |
| European | NAT2, slow | 5.10 | 0.66 | 39.21 | 0.12 |
| Amerindian | Salvador | 0.14 | 0.03 | 0.60 | 0.01 |
| Amerindian | Manaus | 0.46 | 0.19 | 1.12 | 0.01 |
| Amerindian | Sex, female | 1.04 | 0.54 | 2.00 | 0.9 |
| Amerindian | HIV+ | 5.46 | 2.63 | 11.36 | <0.0001 |
| Amerindian | BMI | 1.02 | 0.93 | 1.10 | 0.71 |
| Amerindian | Age | 1.03 | 1.01 | 1.06 | 0.003 |
| Amerindian | Smoke, prior | 1.40 | 0.64 | 3.05 | 0.40 |
| Amerindian | Smoke, current | 1.04 | 0.41 | 2.62 | 0.94 |
| Amerindian | Alcohol, prior | 0.48 | 0.19 | 1.19 | 0.11 |
| Amerindian | Alcohol, current | 0.56 | 0.23 | 1.39 | 0.21 |
| Amerindian | HbA1c | 0.94 | 0.80 | 1.12 | 0.51 |
| Amerindian | DOT, yes | 13.62 | 3.21 | 57.74 | 0.0004 |
| Amerindian | Illicit drug use, prior | 0.92 | 0.40 | 2.10 | 0.84 |
| Amerindian | Illicit drug use, current | 0.75 | 0.24 | 2.39 | 0.63 |
| Amerindian | NAT2, intermediate | 5.13 | 0.66 | 39.77 | 0.12 |
| Amerindian | NAT2, slow | 5.13 | 0.67 | 39.45 | 0.12 |

**Supplemental Table 7:** Covariate results of logistic regression modeling of any grade 3 or higher ADR attributed to TB medication using ancestry proportion as the main predictor.

| Ancestry | Variable | Odds Ratio | Lower CI | Upper CI | P-value |
| --- | --- | --- | --- | --- | --- |
| African | Salvador | --- | --- | --- | --- |
| African | Manaus | 1.15 | 0.43 | 3.11 | 0.78 |
| African | Sex, female | 0.86 | 0.38 | 1.96 | 0.73 |
| African | HIV+ | 4.17 | 1.71 | 10.19 | 0.002 |
| African | BMI | 0.93 | 0.83 | 1.04 | 0.21 |
| African | Age | 1.02 | 0.99 | 1.0 | 0.17 |
| African | Smoke, prior | 1.72 | 0.62 | 4.78 | 0.29 |
| African | Smoke, current | 1.53 | 0.46 | 5.10 | 0.49 |
| African | Alcohol, prior | 0.26 | 0.08 | 0.81 | 0.02 |
| African | Alcohol, current | 0.45 | 0.15 | 1.38 | 0.16 |
| African | HbA1c | 1.05 | 0.89 | 1.30 | 0.61 |
| African | DOT, yes | 14.51 | 1.93 | 109.21 | 0.01 |
| African | Illicit drug use, prior | 0.77 | 0.26 | 2.24 | 0.63 |
| African | Illicit drug use, current | 0.62 | 0.16 | 2.44 | 0.50 |
| African | NAT2, intermediate | 3.55 | 0.44 | 28.49 | 0.23 |
| African | NAT2, slow | 3.35 | 0.42 | 26.53 | 0.25 |
| European | Salvador | --- | --- | --- | --- |
| European | Manaus | 1.11 | 0.45 | 2.75 | 0.82 |
| European | Sex, female | 0.86 | 0.38 | 1.96 | 0.72 |
| European | HIV+ | 4.21 | 1.72 | 10.32 | 0.002 |
| European | BMI | 0.93 | 0.83 | 1.04 | 0.21 |
| European | Age | 1.02 | 0.99 | 1.05 | 0.17 |
| European | Smoke, prior | 1.74 | 0.63 | 4.83 | 0.28 |
| European | Smoke, current | 1.53 | 0.46 | 5.11 | 0.49 |
| European | Alcohol, prior | 0.25 | 0.08 | 0.80 | 0.02 |
| European | Alcohol, current | 0.45 | 0.15 | 1.36 | 0.16 |
| European | HbA1c | 1.05 | 0.86 | 1.29 | 0.63 |
| European | DOT, yes | 14.39 | 1.91 | 108.29 | 0.01 |
| European | Illicit drug use, prior | 0.76 | 0.26 | 2.22 | 0.62 |
| European | Illicit drug use, current | 0.63 | 0.16 | 2.45 | 0.50 |
| European | NAT2, intermediate | 3.54 | 0.44 | 28.39 | 0.23 |
| European | NAT2, slow | 3.36 | 0.42 | 26.60 | 0.25 |
| Amerindian | Salvador | --- | --- | --- | --- |
| Amerindian | Manaus | 1.02 | 0.35 | 2.96 | 0.97 |
| Amerindian | Sex, female | 0.86 | 0.38 | 1.95 | 0.72 |
| Amerindian | HIV+ | 4.22 | 1.72 | 10.35 | 0.002 |
| Amerindian | BMI | 0.93 | 0.83 | 1.04 | 0.20 |
| Amerindian | Age | 1.02 | 0.99 | 1.05 | 0.17 |
| Amerindian | Smoke, prior | 1.74 | 0.63 | 4.84 | 0.29 |
| Amerindian | Smoke, current | 1.54 | 0.46 | 5.13 | 0.49 |
| Amerindian | Alcohol, prior | 0.25 | 0.08 | 0.81 | 0.02 |
| Amerindian | Alcohol, current | 0.45 | 0.15 | 1.38 | 0.16 |
| Amerindian | HbA1c | 1.06 | 0.87 | 1.30 | 0.61 |
| Amerindian | DOT, yes | 14.42 | 1.92 | 108.50 | 0.01 |
| Amerindian | Illicit drug use, prior | 0.76 | 0.26 | 2.22 | 0.62 |
| Amerindian | Illicit drug use, current | 0.63 | 0.16 | 2.46 | 0.51 |
| Amerindian | NAT2, intermediate | 3.51 | 0.44 | 28.16 | 0.24 |
| Amerindian | NAT2, slow | 3.37 | 0.43 | 26.66 | 0.25 |

**Supplemental Table 8:** Covariate results of logistic regression modeling of any hepatic ADR outcome using ancestry proportion as the main predictor.

| Ancestry | Variable | Odds Ratio | Lower CI | Upper CI | P-value |
| --- | --- | --- | --- | --- | --- |
| African | Salvador | 0.34 | 0.10 | 1.11 | 0.07 |
| African | Manaus | 0.52 | 0.19 | 1.39 | 0.19 |
| African | Sex, female | 0.80 | 0.36 | 1.81 | 0.60 |
| African | HIV+ | 4.96 | 2.08 | 11.84 | 0.0003 |
| African | BMI | 1.10 | 1.00 | 1.21 | 0.04 |
| African | Age | 1.0 | 1.00 | 1.06 | 0.04 |
| African | Smoke, prior | 0.57 | 0.23 | 1.39 | 0.22 |
| African | Smoke, current | 0.63 | 0.22 | 1.85 | 0.41 |
| African | Alcohol, prior | 1.44 | 0.44 | 4.72 | 0.54 |
| African | Alcohol, current | 1.48 | 0.45 | 4.87 | 0.52 |
| African | HbA1c | 0.80 | 0.62 | 1.03 | 0.09 |
| African | DOT, yes | 1.35 | 0.58 | 3.12 | 0.49 |
| African | Illicit drug use, prior | 0.97 | 0.38 | 2.44 | 0.95 |
| African | Illicit drug use, current | 0.20 | 0.02 | 1.72 | 0.14 |
| African | NAT2, intermediate | --- | --- | --- | --- |
| African | NAT2, slow | --- | --- | --- | --- |
| European | Salvador | 0.36 | 0.11 | 1.17 | 0.09 |
| European | Manaus | 0.38 | 0.15 | 0.96 | 0.04 |
| European | Sex, female | 0.83 | 0.37 | 1.86 | 0.64 |
| European | HIV+ | 5.14 | 2.15 | 12.28 | 0.0002 |
| European | BMI | 1.20 | 1.00 | 1.20 | 0.05 |
| European | Age | 1.03 | 1.00 | 1.06 | 0.03 |
| European | Smoke, prior | 0.58 | 0.24 | 1.42 | 0.23 |
| European | Smoke, current | 0.64 | 0.22 | 1.88 | 0.42 |
| European | Alcohol, prior | 1.43 | 0.44 | 4.68 | 0.56 |
| European | Alcohol, current | 1.47 | 0.44 | 4.85 | 0.53 |
| European | HbA1c | 0.80 | 0.61 | 1.04 | 0.09 |
| European | DOT, yes | 1.31 | 0.57 | 3.04 | 0.53 |
| European | Illicit drug use, prior | 0.99 | 0.39 | 2.49 | 0.98 |
| European | Illicit drug use, current | 0.21 | 0.03 | 1.79 | 0.15 |
| European | NAT2, intermediate | --- | --- | --- | --- |
| European | NAT2, slow | --- | --- | --- | --- |
| Amerindian | Salvador | 0.39 | 0.12 | 1.24 | 0.11 |
| Amerindian | Manaus | 0.44 | 0.15 | 1.29 | 0.13 |
| Amerindian | Sex, female | 0.80 | 0.36 | 1.80 | 0.59 |
| Amerindian | HIV+ | 5.12 | 2.15 | 12.20 | 0.0002 |
| Amerindian | BMI | 1.10 | 1.00 | 1.20 | 0.05 |
| Amerindian | Age | 1.03 | 1.00 | 1.06 | 0.04 |
| Amerindian | Smoke, prior | 0.57 | 0.23 | 1.40 | 0.22 |
| Amerindian | Smoke, current | 0.64 | 0.22 | 1.87 | 0.41 |
| Amerindian | Alcohol, prior | 1.51 | 0.46 | 4.94 | 0.49 |
| Amerindian | Alcohol, current | 1.59 | 0.48 | 5.26 | 0.44 |
| Amerindian | HbA1c | 0.81 | 0.62 | 1.04 | 0.10 |
| Amerindian | DOT, yes | 1.36 | 0.59 | 3.14 | 0.48 |
| Amerindian | Illicit drug use, prior | 1.02 | 0.41 | 2.56 | 1.00 |
| Amerindian | Illicit drug use, current | 0.21 | 0.02 | 1.76 | 0.101 |
| Amerindian | NAT2, intermediate | --- | --- | --- | --- |
| Amerindian | NAT2, slow | --- | --- | --- | --- |

**Supplemental Table 9:** Covariate results of logistic regression modeling of hepatic ADR attributed to TB medications using ancestry proportion as the main predictor.

| Ancestry | Variable | Odds Ratio | Lower CI | Upper CI | P-value |
| --- | --- | --- | --- | --- | --- |
| African | Salvador | 0.42 | 0.12 | 1.39 | 0.15 |
| African | Manaus | 0.46 | 0.16 | 1.28 | 0.14 |
| African | Sex, female | 0.78 | 0.33 | 1.84 | 0.57 |
| African | HIV+ | 5.19 | 2.10 | 12.84 | 0.0004 |
| African | BMI | 1.09 | 0.99 | 1.20 | 0.08 |
| African | Age | 1.03 | 1.00 | 1.06 | 0.02 |
| African | Smoke, prior | 0.60 | 0.24 | 1.49 | 0.27 |
| African | Smoke, current | 0.44 | 0.13 | 1.51 | 0.19 |
| African | Alcohol, prior | 1.48 | 0.45 | 4.90 | 0.52 |
| African | Alcohol, current | 1.25 | 0.36 | 4.28 | 0.73 |
| African | HbA1c | 0.76 | 0.57 | 1.01 | 0.06 |
| African | DOT, yes | 1.23 | 0.52 | 2.88 | 0.64 |
| African | Illicit drug use, prior | 0.92 | 0.35 | 2.43 | 0.86 |
| African | Illicit drug use, current | 0.30 | 0.04 | 2.57 | 0.27 |
| African | NAT2, intermediate | --- | --- | --- | --- |
| African | NAT2, slow | --- | --- | --- | --- |
| European | Salvador | 0.44 | 0.13 | 1.46 | 0.18 |
| European | Manaus | 0.37 | 0.14 | 0.97 | 0.04 |
| European | Sex, female | 0.80 | 0.34 | 1.87 | 0.60 |
| European | HIV+ | 5.35 | 2.16 | 13.23 | 0.0003 |
| European | BMI | 1.09 | 0.99 | 1.20 | 0.09 |
| European | Age | 1.03 | 1.00 | 1.06 | 0.02 |
| European | Smoke, prior | 0.61 | 0.24 | 1.50 | 0.28 |
| European | Smoke, current | 0.45 | 0.13 | 1.53 | 0.20 |
| European | Alcohol, prior | 1.49 | 0.45 | 4.94 | 0.51 |
| European | Alcohol, current | 1.26 | 0.37 | 4.36 | 0.71 |
| European | HbA1c | 0.76 | 0.57 | 1.02 | 0.07 |
| European | DOT, yes | 1.21 | 0.51 | 2.84 | 0.66 |
| European | Illicit drug use, prior | 0.94 | 0.36 | 2.48 | 0.90 |
| European | Illicit drug use, current | 0.31 | 0.04 | 2.66 | 0.29 |
| European | NAT2, intermediate | --- | --- | --- | --- |
| European | NAT2, slow | --- | --- | --- | --- |
| Amerindian | Salvador | 0.45 | 0.14 | 1.48 | 0.19 |
| Amerindian | Manaus | 0.44 | 0.14 | 1.36 | 0.16 |
| Amerindian | Sex, female | 0.77 | 0.33 | 1.82 | 0.56 |
| Amerindian | HIV+ | 5.26 | 2.13 | 12.99 | 0.0003 |
| Amerindian | BMI | 1.09 | 0.97 | 1.20 | 0.09 |
| Amerindian | Age | 1.03 | 1.00 | 1.06 | 0.03 |
| Amerindian | Smoke, prior | 0.60 | 0.24 | 1.49 | 0.27 |
| Amerindian | Smoke, current | 0.44 | 0.13 | 1.51 | 0.19 |
| Amerindian | Alcohol, prior | 1.56 | 0.47 | 5.13 | 0.47 |
| Amerindian | Alcohol, current | 1.33 | 0.39 | 4.57 | 0.65 |
| Amerindian | HbA1c | 0.76 | 0.57 | 1.02 | 0.07 |
| Amerindian | DOT, yes | 1.24 | 0.53 | 2.92 | 0.62 |
| Amerindian | Illicit drug use, prior | 0.95 | 0.36 | 2.50 | 0.92 |
| Amerindian | Illicit drug use, current | 0.30 | 0.04 | 2.59 | 0.27 |
| Amerindian | NAT2, intermediate | --- | --- | --- | --- |
| Amerindian | NAT2, slow | --- | --- | --- | --- |

**Supplemental Table 10:** Covariate results of logistic regression modeling of TB treatment failure or recurrence using ancestry proportion as the main predictor.

| Ancestry | Variable | Odds Ratio | Lower CI | Upper CI | P-value |
| --- | --- | --- | --- | --- | --- |
| African | Salvador | 2.83 | 0.95 | 8.47 | 0.06 |
| African | Manaus | 7.11 | 2.38 | 21.20 | 0.0004 |
| African | Sex, female | 1.80 | 0.86 | 3.76 | 0.12 |
| African | HIV+ | 1.12 | 0.46 | 2.70 | 0.80 |
| African | BMI | 0.98 | 0.89 | 1.09 | 0.72 |
| African | Age | 0.97 | 0.96 | 1.02 | 0.37 |
| African | Smoke, prior | 1.02 | 0.42 | 2.49 | 0.97 |
| African | Smoke, current | 1.67 | 0.56 | 4.99 | 0.36 |
| African | Alcohol, prior | 1.44 | 0.43 | 4.87 | 0.56 |
| African | Alcohol, current | 1.01 | 0.29 | 3.53 | 0.99 |
| African | HbA1c | 1.16 | 0.99 | 1.37 | 0.06 |
| African | DOT, yes | 0.48 | 0.24 | 0.97 | 0.04 |
| African | Illicit drug use, prior | 0.84 | 0.31 | 2.23 | 0.72 |
| African | Illicit drug use, current | 1.03 | 0.30 | 3.61 | 0.96 |
| African | NAT2, intermediate | 0.59 | 0.19 | 1.77 | 0.35 |
| African | NAT2, slow | 0.77 | 0.27 | 2.22 | 0.63 |
| European | Salvador | 3.12 | 1.05 | 9.23 | 0.04 |
| European | Manaus | 6.82 | 2.43 | 19.18 | 0.0003 |
| European | Sex, female | 1.82 | 0.87 | 3.80 | 0.11 |
| European | HIV+ | 1.13 | 0.47 | 2.72 | 0.79 |
| European | BMI | 0.98 | 0.89 | 1.09 | 0.72 |
| European | Age | 0.99 | 0.96 | 1.02 | 0.36 |
| European | Smoke, prior | 1.01 | 0.42 | 2.48 | 0.97 |
| European | Smoke, current | 1.72 | 0.55 | 5.14 | 0.33 |
| European | Alcohol, prior | 1.48 | 0.44 | 5.00 | 0.52 |
| European | Alcohol, current | 1.02 | 0.29 | 3.56 | 0.97 |
| European | HbA1c | 1.16 | 0.99 | 1.37 | 0.06 |
| European | DOT, yes | 0.49 | 0.24 | 0.98 | 0.04 |
| European | Illicit drug use, prior | 0.83 | 0.31 | 2.23 | 0.71 |
| European | Illicit drug use, current | 1.03 | 0.29 | 3.60 | 0.97 |
| European | NAT2, intermediate | 0.58 | 0.19 | 1.76 | 0.34 |
| European | NAT2, slow | 0.76 | 0.26 | 2.21 | 0.61 |
| Amerindian | Salvador | 2.88 | 0.99 | 8.41 | 0.05 |
| Amerindian | Manaus | 8.50 | 2.74 | 26.35 | 0.0002 |
| Amerindian | Sex, female | 1.81 | 0.87 | 3.78 | 0.11 |
| Amerindian | HIV+ | 1.11 | 0.46 | 2.68 | 0.81 |
| Amerindian | BMI | 0.98 | 0.89 | 1.09 | 0.73 |
| Amerindian | Age | 0.99 | 0.96 | 1.02 | 0.35 |
| Amerindian | Smoke, prior | 1.02 | 0.42 | 2.48 | 0.97 |
| Amerindian | Smoke, current | 1.70 | 0.58 | 5.05 | 0.34 |
| Amerindian | Alcohol, prior | 1.46 | 0.43 | 4.92 | 0.54 |
| Amerindian | Alcohol, current | 1.00 | 0.29 | 3.49 | 1.00 |
| Amerindian | HbA1c | 1.16 | 0.99 | 1.36 | 0.06 |
| Amerindian | DOT, yes | 0.49 | 0.24 | 0.98 | 0.04 |
| Amerindian | Illicit drug use, prior | 0.81 | 0.30 | 2.18 | 0.68 |
| Amerindian | Illicit drug use, current | 1.01 | 0.29 | 3.52 | 0.98 |
| Amerindian | NAT2, intermediate | 0.58 | 0.19 | 1.75 | 0.33 |
| Amerindian | NAT2, slow | 0.76 | 0.26 | 2.19 | 0.61 |

**Supplemental Table 11.** Grade 2+ adverse drug reaction (ADR) related to tuberculosis treatment modeled stratified by HIV status.

1. HIV Negative

| Ancestry | Unadjusted | | | Adjusted | | |
| --- | --- | --- | --- | --- | --- | --- |
|  | **OR (95% CI)** | **P-value** | **Pseudo-R^2^** | **OR (95% CI)** | **P-value** | **Pseudo-R^2^** |
| African | 0.21 (0.09 - 0.47) | 1x10^-4^ | 0.05 | 0.25 (0.10 - 0.64) | 0.004 | 0.24 |
| European | 4.27 (1.87 - 9.76) | 6x10^-4^ | 0.04 | 3.06 (1.23 - 7.80) | 0.02 | 0.23 |
| Amerindian | 1.77 (0.62 - 5.09) | 0.29 | 0.003 | 1.66 (0.41 - 6.78) | 0.48 | 0.21 |

1. HIV Positive

| Ancestry | Unadjusted | | | Adjusted | | |
| --- | --- | --- | --- | --- | --- | --- |
|  | **OR (95% CI)** | **P-value** | **Pseudo-R^2^** | **OR (95% CI)** | **P-value** | **Pseudo-R^2^** |
| African | 2.74 (0.78 - 9.58) | 0.12 | 0.02 | 1.01 (0.20 - 5.01) | 0.99 | 0.21 |
| European | 1.42 (0.45 - 4.47) | 0.54 | 0.003 | 1.38 (0.41 - 4.63) | 0.60 | 0.21 |
| Amerindian | 0.38 (0.13 - 1.06) | 0.06 | 0.04 | 0.65 (0.17 - 2.49) | 0.53 | 0.21 |

OR: odds ratio; CI: confidence interval; ADR: adverse drug reaction; TB: Tuberculosis; Ancestry proportion was used as the main predictor in logistic regression modeling and adjusted models contained the covariates *NAT2* acetylator status, BMI, age, HbA1c, sex, DOT status, recruitment site, and smoking, alcohol, and drug usage. Odds ratios are given for 10% increase in ancestry proportion.
